# 

**Published:** 1893-09

**Authors:** 


					CONTENTS.
Th
Increase of Insanity.
dv p tuurcaac ui luaauitjf*
i? b ? S.W. Cobbold, M.D.Wiirz.,
'R-C.P. Ed
143
? *-*u? ??? ... ... ???
eT?hmbination of Syphilis and
rta??rcul?sis, especially in re-
L ? to Laryngeal Affections.
Bv t> ? laryngeal Affections.
Lond ATSON Williams, M.D.
155
5 y*e Cauda Equina: Opera-
tif?^ tC c ^uaa equina.: u
p Relief of Symptoms.
ByJ.
|v o ui oy inpiuiiib. uj j.
Biic J"' M.B.Edin., and J. Paul
^ush, M.R.C.s, Eng. ... ...
161
M
Hn: a Summary.
By John
M : ? ?? summary, ay joiin
Meredith, M.D.Ed.
171
Progress of the Medical Sciences:
Medicine.
By George Parker,
M.D.
175
Surgery.
By W. J. Penny
180
Laryngology.
By B. J. Baron,
187
Reviews of Books
190
Notes on Preparations for the
Sick
210
The Library of the Bristol Medico-
Chirurgleal Society
212
Scraps
215

				

